# Adipose‐derived mesenchymal stem cells overexpressing prion improve outcomes via the NLRP3 inflammasome/DAMP signalling after spinal cord injury in rat

**DOI:** 10.1111/jcmm.17620

**Published:** 2023-01-20

**Authors:** Tsung‐Cheng Yin, Yi‐Chen Li, Pei‐Hsun Sung, John Y. Chiang, Pei‐Lin Shao, Hon‐Kan Yip, Mel S. Lee

**Affiliations:** ^1^ Department of Orthopaedic Surgery Kaohsiung Chang Gung Memorial Hospital, College of Medicine, Chang Gung University Kaohsiung Taiwan; ^2^ Center for General Education Cheng Shiu University Kaohsiung Taiwan; ^3^ Clinical Medicine Research Center National Cheng Kung University Hospital, College of Medicine, National Cheng Kung University Tainan Taiwan; ^4^ Center of Cell Therapy National Cheng Kung University Hospital, College of Medicine, National Cheng Kung University Tainan Taiwan; ^5^ Institute of Clinical Medicine College of Medicine National Cheng Kung University Tainan Taiwan; ^6^ Division of Cardiology, Department of Internal Medicine Kaohsiung Chang Gung Memorial Hospital, College of Medicine, Chang Gung University Kaohsiung Taiwan; ^7^ Center for Shockwave Medicine and Tissue Engineering Kaohsiung Chang Gung Memorial Hospital Kaohsiung Taiwan; ^8^ Institute for Translational Research in Biomedicine Kaohsiung Chang Gung Memorial Hospital Kaohsiung Taiwan; ^9^ Department of Computer Science & Engineering National Sun Yat‐sen University Kaohsiung Taiwan; ^10^ Department of Healthcare Administration and Medical Informatics Kaohsiung Medical University Kaohsiung Taiwan; ^11^ Department of Nursing Asia University Taichung Taiwan; ^12^ Department of Medical Research China Medical University Hospital, China Medical University Taichung Taiwan; ^13^ Division of Cardiology, Department of Internal Medicine Xiamen Chang Gung Hospital Xiamen China; ^14^ Department of Orthopedic Surgery Pao‐Chien Hospital Pingtung Taiwan

**Keywords:** adipose‐derived mesenchymal stem cells, inflammasome, inflammatory signalling, prion protein, spinal cord injury

## Abstract

Traumatic spinal cord injury (SCI) is a highly destructive disease in human neurological functions. Adipose‐derived mesenchymal stem cells (ADMSCs) have tissue regenerations and anti‐inflammations, especially with prion protein overexpression (PrPc^OE^). Therefore, this study tested whether PrPc^OE^‐ADMSCs therapy offered benefits in improving outcomes via regulating nod‐like‐receptor‐protein‐3 (NLRP3) inflammasome/DAMP signalling after acute SCI in rats. Compared with ADMSCs only, the capabilities of PrPc^OE^‐ADMSCs were significantly enhanced in cellular viability, anti‐oxidative stress and migration against H_2_O_2_ and lipopolysaccharide damages. Similarly, PrPc^OE^‐ADMSCs significantly inhibited the inflammatory patterns of Raw264.7 cells. The SD rats (*n* = 32) were categorized into group 1 (Sham‐operated‐control), group 2 (SCI), group 3 (SCI + ADMSCs) and group 4 (SCI + PrPc^OE^‐ADMSCs). Compared with SCI group 2, both ADMSCs and PrPc^OE^‐ADMSCs significantly improved neurological functions. Additionally, the circulatory inflammatory cytokines levels (TNF‐α/IL‐6) and inflammatory cells (CD11b/c+/MPO+/Ly6G+) were highest in group 2, lowest in group 1, and significantly higher in group 3 than in group 4. By Day 3 after SCI induction, the protein expressions of inflammasome signalling (HGMB1/TLR4/MyD88/TRIF/c‐caspase8/FADD/p‐NF‐κB/NEK7/NRLP3/ASC/c‐caspase1/IL‐ß) and by Day 42 the protein expressions of DAMP‐inflammatory signalling (HGMB1/TLR‐4/MyD88/TRIF/TRAF6/p‐NF‐κB/TNF‐α/IL‐1ß) in spinal cord tissues displayed an identical pattern as the inflammatory patterns. In conclusion, PrPc^OE^‐ADMSCs significantly attenuated SCI in rodents that could be through suppressing the inflammatory signalling.

## 1 INTRODUCTION

Traumatic spinal cord injury (SCI) is a highly destructive disease that results in deficits in human neurological functions.^1^, ^2^ The global incidence of acute SCI is approximately 10 cases per 100,000 persons, resulting in over 700,000–800,000 new patients diagnosed annually worldwide.^2^, ^3^, ^4^ Regrettably, despite advances in medical and surgical cares, the current clinical therapies for this devastating disease are primarily ineffective.^5^, ^6^, ^7^, ^8^, ^9^ When acute SCI develops, it is generally considered to progress in two stages. The primary injury is the mechanical damage caused by a direct external force. It is followed by secondary injury, which refers to the delayed spread of damage brought about by factors such as inflammatory cytokines, tissue acidosis, glutamate, and dysregulation of the electrolyte homeostasis, leading to further functional deterioration.^10^, ^11^ Additionally, studies have further demonstrated that the secondary damage of SCI is orchestrated by the various pathophysiologic mechanisms, including inflammatory reaction,^12^ mitochondrial dysfunction^13^ and oxidative stress.^14^ However, the detailed molecular mechanisms underlying SCI are currently not completely understood.

An inflammasome is now clearly defined by its sensor protein [i.e. a pseudo‐response regulator (PRR)], which oligomerizes to form a pro‐caspase‐1 activating platform in response to DAMPs. There are four members of PRRs that have been identified to form inflammasomes, including nucleotide‐binding oligomerization domain (NOD), leucine‐rich repeat (NLR)‐containing proteins (NLRP) family members NLRP1, NLRP3 and NLRC4.^15^, ^16^, ^17^ The NLRP3 inflammasome pathway has been the most extensively investigated, and the results showed that dysregulation of this signalling is closely related to the development of many human diseases, such as neuroinflammation, metabolic inflammation and immune inflammation.^18^

Some studies have previously demonstrated that acute SCI triggers NLRP3 inflammasome activation in spinal cord tissue^19^, ^20^, ^21^ and spinal cord microglia. Additionally, NLRP3 was identified in neurons, microglia and astroglia, and microglia was the major contributors to the spinal cord damage in the setting of acute SCI.^21^ These findings^15^, ^16^, ^17^, ^18^, ^19^, ^20^, ^21^ raise the hypothesis that to develop a new strategic management that specifically inhibits the generation of NLRP3 inflammasome in the setting of acute SCI may be a fundamental issue for the treatment of SCI patients, especially in those who are refractory to conventional therapy.

Plentiful data from clinical trials and experimental studies have shown that mesenchymal stem cell (MSC) therapy, especially those of adipose‐derived mesenchymal stem cells (ADMSCs) have the capability of ameliorating inflammatory reaction^22^, ^23^, ^24^ and suppressing the innate and adaptive immunity^23^, ^25^, ^26^ through downregulating the immunogenicity.^23^, ^25^, ^26^, ^27^, ^28^, ^29^, ^30^ Our preclinical studies have shown that ADMSCs have a strong capacity for immunomodulation that significantly reduced post‐heart transplanted acute rejection,^31^ and effectively suppressed both overwhelming inflammatory and inflammatory‐immune reactions,^23^, ^25^, ^30^, ^31^, ^32^, ^33^ resulting in an improvement of prognostic outcomes. Recently, our study has demonstrated that valsartan therapy preserved residual renal function in chronic kidney disease (CKD) rats mainly through upregulating the cellular prion protein (PrPc) expression.^34^ Intriguingly, our more recent study has demonstrated that overexpression of PrPc (PrPc^OE^) in ADMSCs has a strong capacity for downregulating the inflammation and oxidative stress and augmentation of myocardial regeneration.^35^ Surprisingly, there has been yet no data to address the impact of PrPc^OE^ in ADMSCs on protecting the spinal cord in the setting of acute SCI. Accordingly, we tested the hypothesis that PrPc^OE^ in ADMSCs (i.e. called PrPc^OE^‐ADMSCs) could offer neuroprotective and neurogenerative effects by suppressing NLRP3 inflammasome and signalling in SCI rats.

## 2 MATERIALS AND METHODS

### 2.1 Ethics

All animal procedures were approved by the Institute of Animal Care and Use Committee at Kaohsiung Chang Gung Memorial Hospital (Affidavit of Approval of Animal Use Protocol No. 2020061706), followed the Guide for the Care and Use of Laboratory Animals, and housed in an Association for Assessment and Accreditation of Laboratory Animal Care International (AAALAC)‐approved animal facility in our institutes with controlled temperature and light cycles (24°C and 12/12 light cycle).

### 2.2 Procedure and protocol of SCI in Sprague–Dawley rats and animal grouping

Pathogen‐free, adult‐male Sprague–Dawley rats (*n* = 32) weighing 300–325 g (Charles River Technology, BioLASCO Taiwan Co. Ltd) were used for the present study. Animals in the SC group underwent opening of the skin and muscle layers only, while acute traumatic SCI groups received a complete traumatic SCI procedure. For SCI induction, animals were anaesthetised with inhalational 2.0% isoflurane and placed prone position on a warming pad at 37°C for laminectomy at T10 to T12. A modified aneurysm clip was further used to create an impact‐compression injury of the spinal cord, and then, it was left for compressing the spinal cord for 30 s before removal.

The animals were categorized into group 1 [sham‐operated control (SC)], group 2 SCI only +200 μl culture medium by peri‐spinal cord injury area injection and intravenous injection], group 3 [SCI + ADMSCs (1.5 × 10^6^ cells in total, categorized into 3.0 × 10^5^ cells by peri‐spinal cord injury area injection and 1.2 × 10^6^ by intravenous injection 3 h after SCI procedure, respectively), and group 4 [SCI + PrPc^OE^‐ADMSCs (1.5 × 10^6^ cells in total, categorized into 3.0 × 10^5^ cells by peri‐spinal cord injury area injection and 1.2 × 10^6^ by intravenous injection 3 h after SCI procedure, respectively)]. Neurological status [i.e. by Basso, Beattie, Bresnahan (BBB) functional scale] was assessed prior to and by Days 1, 3, 7, 14, 21, 35 and 42 after the traumatic SCI procedure.

### 2.3 Cell culture

Raw 264.7 (BCRC 60001) were cultured in Dulbecco's modified Eagle's medium with high glucose (Gibco) containing 10% Fetal Bovine Serum (Gibco), 100 μg/ml streptomycin and 100 units/ml penicillin (Gibco).

### 2.4 Isolation of adipose tissue for culturing ADMSCs

For the preparation of allogenic ADMSCs, additional 12 rats were utilized in the present study. The procedure and protocol for ADMSCs isolation and culturing have been described in our previous reports.^22^, ^24^, ^25^, ^26^ In detail, allogenic ADMSC donated rats were anaesthetised and then cut into <1 mm^3^ size pieces using a pair of sharp, sterile surgical scissors. After serial incubation and centrifugation, the cells were obtained and cultured in a 100 mm diameter dish with 10 ml DMEM culture medium containing 10% FBS for 14 days.

### 2.5 Transfection of ADMSCs with plasmids for cellular prion protein (PrPc) overexpression

The procedure and protocol have been reported in our recent study.^35^ We purchased the pCS6‐PRNP plasmid from Transomic Technologies . The plasmid transfection process was carried out with Lipofectamine 3000 according to the steps specified in the manual. The steps were briefly described as follows: 10 μg PRNP expression vector and 20 μl Lipofectamine 3000 were first incubated at room temperature for 15 minutes, followed by overnight incubation of cells at 37°C in a humidified atmosphere of 5% CO_2_ and Lipofectamine (i.e. mixed them together), and relevant experiments were carried out.

### 2.6 MTT assay

5 × 10^3^ ADMSC cells were seeded in 96‐well dishes. After the PRNP expression vector was transfected overnight, the cells were treated with 150 μM H_2_O_2_ or 50 ng/ml lipopolysaccharide (LPS) (Sigma). After 6 h incubation, the medium was replaced and replenished, followed by incubation for an additional 24–72 h. At the time point for detection, the medium was removed, and 200 μl MTT reagent was added to the cells for 30 min. Following the formed formazan was solubilized by DMSO in the cell, the optical density was detected at wavelength 595 nm with a spectrophotometer.

### 2.7 Transwell migration assay for assessing the migratory capacity of PrPc^OE^‐ADMSCs

The 3.5 × 10^4^ cells were then added to the Boyden chambers (8 μm pore size; Millipore) with 0.5% FBS containing medium, and assay media containing 10% FBS was added to the culture plates. After 24 h incubation, the nonmotile cells at the top of the filter were removed, and the motile cells at the bottom of the filter were fixed with methanol and stained with a one‐tenth dilution of Giemsa (Sigma Corporation). The number of migrated cells in each chamber was counted in five randomly chosen fields under the microscope for three independent experiments.

### 2.8 Cell culturing for ADMSCs or Raw 264.7 cells

5 × 10^5^ ADMSCs were seeded overnight to the 12‐well Boyden chambers (8 um pore size; Millipore). After the PRNP expression vector was transfected for 24 h, the ADMSCs were treated with 150 μM H₂O_2_ or 50 ng/ml LPS (Sigma) for 6 hours. After the medium was exchanged and replenished, the cells were collected for western blotting and immunofluorescence stain following an additional 24 h incubation.

2 × 10^5^ ADMSCs were seeded to the 6‐well Boyden chambers (8 μm pore size; Millipore) overnight. Following the PRNP expression vector transfected for an additional 24 h, 8 × 10^5^ Raw 264.7 cells were seeded on the coverslips in a 6‐well dish for 24 h. After 50 ng/ml LPS stimulation (Sigma) for 6 h, the medium was exchanged and covered with the Boyden chambers, which the PrPc protein was over‐expressed in the ADMSC cells. After an additional incubation for 24 h, Raw 264.7 cells were analysed by immunofluorescence assay and western blot with indicated antibodies.

### 2.9 Procedure and protocol of flow cytometry and ELISA

After the cells were rinsed twice with PBS, they were incubated with a serum‐free medium containing 10 μM H_2_DCFDA in a 37°C incubator for 20 min. Following by rinsing twice with PBS to remove residue H_2_DCFDA, the cells were incubated with a serum‐containing culture medium for an additional 30 min. Then, the cells were analysed by flow cytometry. To analyse the content of superoxide radicals in the mitochondria, the cells were incubated in PBS containing 5 μM mitoSOX for 10 min and analysed the fluorescence intensity by flow cytometry.

To verify the circulating level of Ly6G+, CD11b/c + or myeloperoxidase (MPO) + cell populations, whole blood cells were stained and performed by flow cytometric analysis (FC500, Beckman) on Days 3 and 7 after SCI induction. The 1.0 × 10^6^ cells were stained with Ly6g‐Alexa Fluor 488 (Abcam), CD11b/c‐PE (BD bioscience), or MPO‐PE (Abcam) antibodies to detect cell surface markers.

To analyse circulatory levels of inflammatory biomarkers, serum tumour necrosis factor (TNF)‐α and interleukin (IL)‐6 concentrations were assessed by 6H and 72 h after SCI induction in duplicate with a commercial ELISA kit (R&D Systems).

### 2.10 Basso, Beattie, Bresnahan (BBB) functional scale for the assessment of the locomotor capacity of rats after acute SCI

The animals were followed for 6 weeks (i.e. 42 days) after the acute SCI procedure. The procedure and protocol of the BBB scale were according to the previous reports.^36^, ^37^ Briefly, each rat was placed in an 80 × 80 × 30 cm^3^ clear box lined with a blue non‐slippery material and stimulated to move freely. All movements were recorded by video. Identical copies of the edited videos were monitored by two independent evaluators blinded to the degree of injury severity. Each evaluator accessed the locomotor capacity of rats using the BBB functional scale. At the end of the experiments, the parameters in each rat were calculated and averaging scores from these two evaluators.

### 2.11 Western blot analysis

Western blot analysis was performed as described previously.^22^, ^23^, ^24^ Equal amounts (50 μg) of protein extracts were loaded and separated by SDS‐PAGE and transferred to PVDF membranes. Next, the membranes were incubated with the indicated primary antibodies as Table S1 for 1 h at room temperature. Horseradish peroxidase‐conjugated anti‐rabbit IgG (1:2000, Cell Signalling) was used as a secondary antibody. Immuno‐reactive bands were visualized by enhanced chemiluminescence (ECL; Amersham Biosciences) and digitized using Labwork software (UVP).

### 2.12 Immunofluorescent (IF) staining

IF staining proceeded as we previously reported.^22^, ^23^, ^24^ Rehydrated paraffin sections were first treated with 3% H_2_O_2_ for 30 min and then incubated with primary antibodies specifically against CD11b/c and CD68. Three sections of spinal‐cord specimens were analysed in each rat. Three randomly selected high‐power fields (HPFs) were analysed per section for quantification. Finally, the mean number of positively stained cells per HPF for each animal was determined across all nine HPFs.

### 2.13 Statistical analysis

Quantitative data are expressed as mean ± SD. The statistical analysis was performed by anova followed by Bonferroni multiple comparison post‐hoc test. We utilized SAS statistical software for Windows version 8.2 (SAS Institute). A probability value <0.05 was considered statistically significant.

## 3 RESULTS

### 3.1 Impact of PrPc^OE^‐ADMSCs on time courses of cell viability and anti‐oxidative stress underwent oxidative stress and inflammatory stimulation in vitro

**FIGURE 1:**
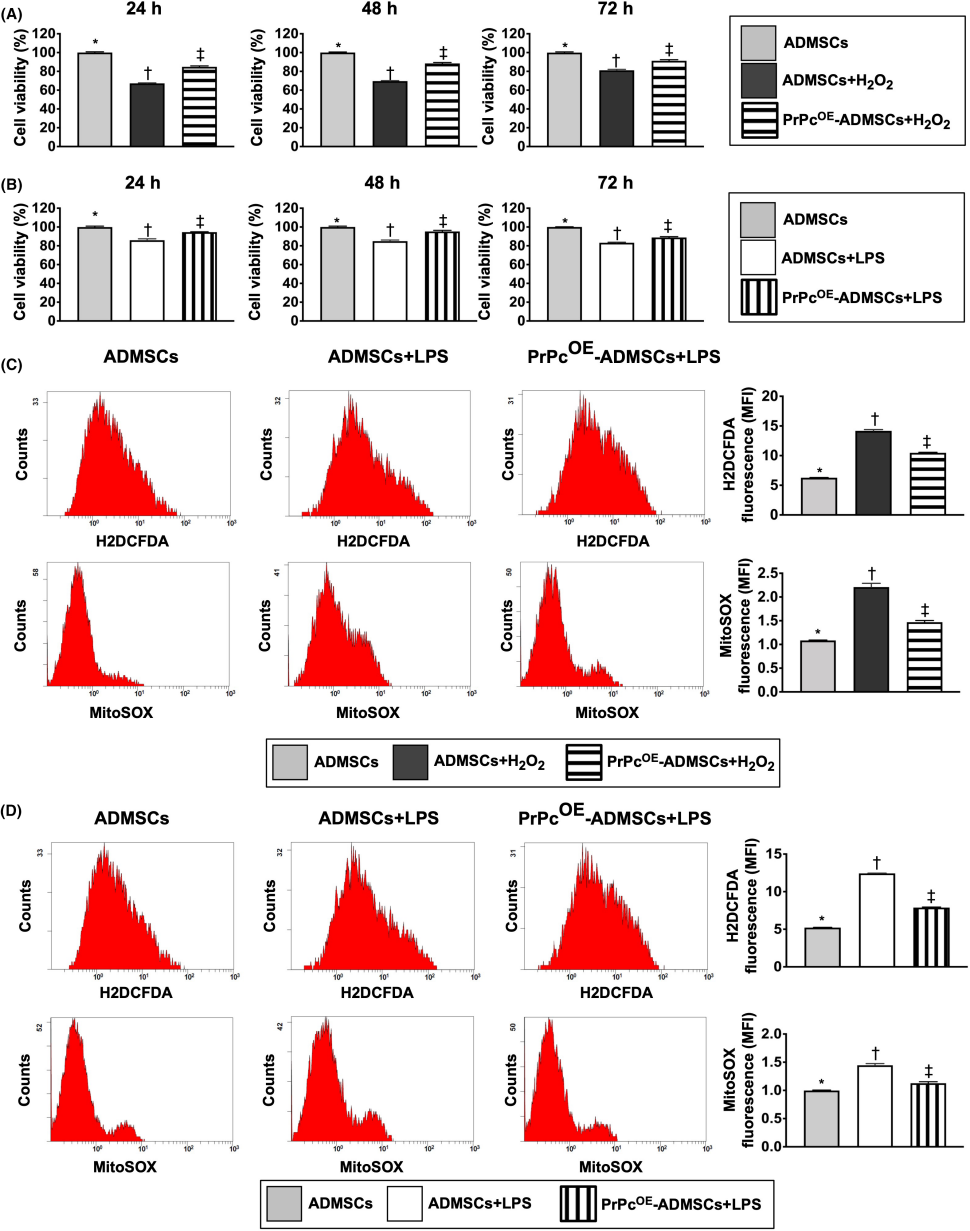
Impact of PrPc^OE^‐ADMSCs in cell viability and anti‐oxidative stress under the condition of oxidative stress and inflammatory stimulation on time courses. (A) Cell viability in the condition of oxidative stress (i.e. H_2_O_2_ treatment) at 24 h, 48 h and 72 h. * vs. other groups with different symbols (†, ‡), *p* < 0.0001. (B) Cell viability in the condition of inflammatory stimulation (i.e., LPS treatment) 24 h, 48 h, and 72 h. (C) Illustrating the flow cytometric analysis for identification of fluorescent intensities u of total intracellular reactive oxygen species (ROS) by H_2_DCFDA staining and mitochondrial ROS by MitoSOX staining under the condition of H_2_O_2_ treatments. Analytical result of mean fluorescent intensity (MFI) of total intracellular ROS or mitochondrial ROS were shown in right parts. (D) Illustrating the flow cytometric analysis for identification of fluorescent intensities u of total ROS by H_2_DCFDA staining and mitochondrial ROS by MitoSOX staining under the condition of LPS treatments. Analytical result of mean fluorescent intensity (MFI) of total intracellular ROS or mitochondrial ROS were shown in right parts. * vs. other groups with different symbols (†, ‡), *p* < 0.0001. Symbols (*, †, ‡) indicate significance for each other (at 0.05 level). All statistical analyses were performed by one‐way anova, followed by Bonferroni multiple comparison post hoc tests (*n* = 6 for each group). H_2_O_2_, hydrogen peroxide; ADMSCs, adipose‐derived mesenchymal stem cells; PrPc^OE^‐ADMSCs, overexpression of cellular prion protein in ADMSCs; LPS, lipopolysaccharide

First, to verify the protective capacity of PrPc^OE^ in ADMSCs (i.e. PrPc^OE^‐ADMSCs) on cell viability against oxidative stress or inflammatory stimulation, the in vitro study was categorized into G1 (ADMSCs only), G2 (ADMSCs + H_2_O_2_) and G3 (PrPc^OE^‐ADMSCs + H_2_O_2_), respectively. The result of the MTT assay demonstrated that as compared with G1, the cell viability at the time intervals of 24, 48 and 72 h was significantly lower in G2 and was significantly reversed in G3 (Figure 1A).

Second, to further verify whether PrPc^OE^‐ADMSCs also had the capacity to protect the cells against the inflammatory stress, the in vitro study was categorized into G4 (ADMSCs only), G5 [ADMSCs + lipopolysaccharide (LPS)] and G6 (PrPc^OE^‐ADMSCs + LPS), respectively. The MTT assay result showed that compared with G4 the cell viability at the time points of 24, 48 and 72 h was significantly lower in G5 than was significantly reversed in G6 (Figure 1B).

Accordingly, the results of these in vitro studies implicated that PrPc^OE^‐ADMSCs offered much more excellent benefits than the ADMSCs‐only counterpart in maintaining the cell proliferation against oxidative stress or inflammatory inhibition of cell growth.

To verify these issues of oxidative stress and inflammation, the in vitro study was categorized into six groups as shown in Figure 1A,B, that is G1 to G6 and the flow cytometric analysis was utilized in the present study. The result showed that the fluorescent intensity of total intracellular and mitochondrial ROS under H_2_O_2_ treatment were significantly increased in G2 than in G1 and G3 and significantly increased in G3 than in G1 (Figure 1C). Additionally, under LPS treatment these parameters were significantly increased in G5 than in G4 and G6, and significantly increased in G6 than in G4 (Figure 1D). Our findings supported that PrPc gene overexpression in ADMSCs enhanced the resistant capacity against the generation of ROS resulted from oxidative stress and inflammatory stimulation.

### 3.2 Impact of PrPc^OE^‐ADMSCs on cell migration under oxidative stress and inflammatory stimulations

To elucidate whether PrPc^OE^‐ADMSC treatment would preserve the ADMSCs migratory ability with respect to oxidative stress and inflammatory stimulations, the in vitro study was categorized into six groups as shown in Figure 1.

**FIGURE 2:**
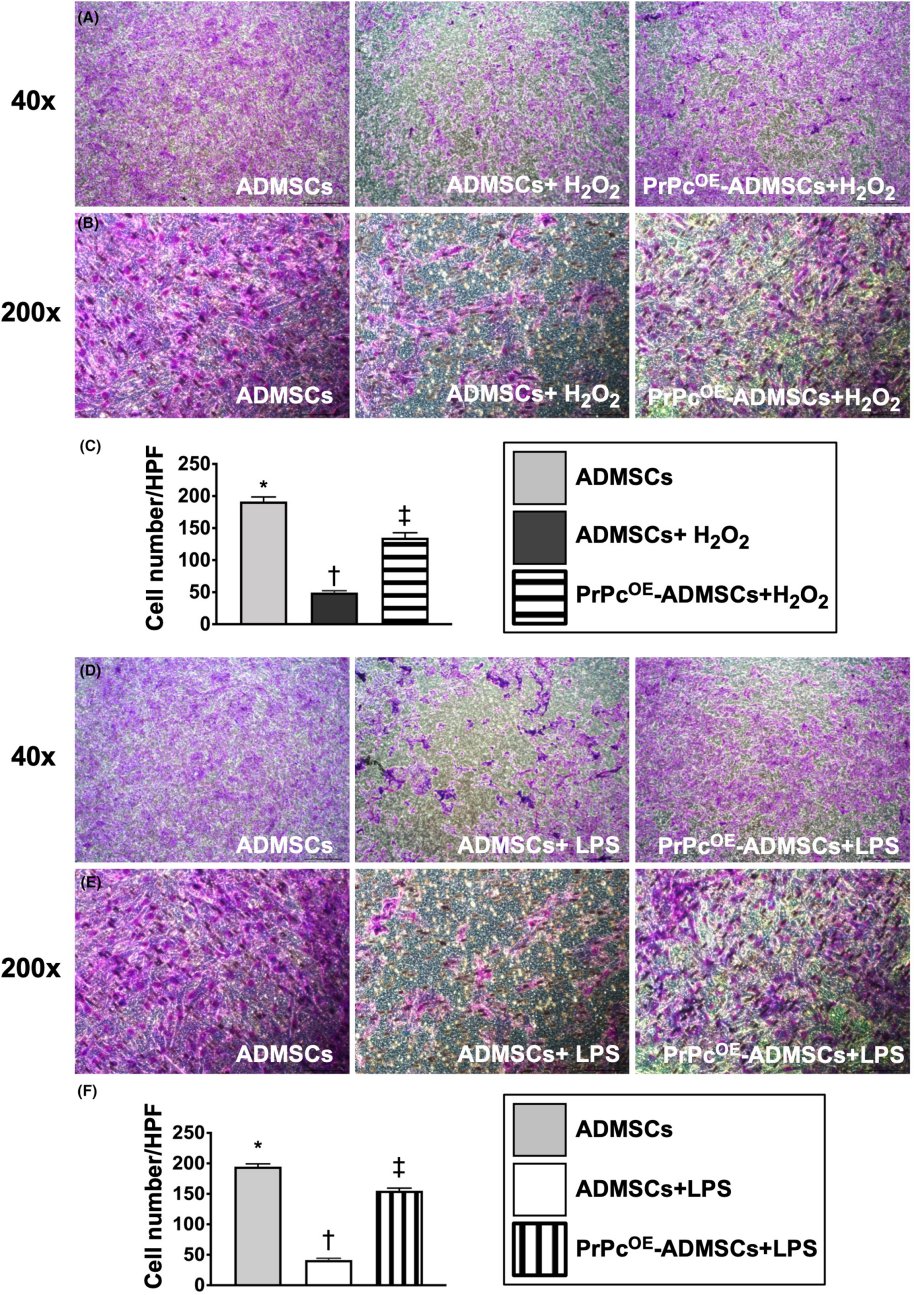
Impact of PrPc^OE^‐ADMSCs on cell migration under oxidative stress and inflammatory stimulation. (A,B) Illustrating the microscopic finding, that is 40x (A) and 200x (B), for identification of migratory cell ability (pink colour) in the condition of oxidative stress, scale bar over the right lower corner in 200x manifestation was 50 μM. (C) Migratory cell number, * vs. other groups with different symbols (†, ‡), *p* < 0.0001. (D‐E) Illustrating the microscopic finding, that is 40x (D) and 200x (E), for identification of migratory cell ability (pink colour) in the condition of inflammatory stimulation, scale bar over the right lower corner in 200x manifestation was 50 μM. (F) Migratory cell number, * vs. other groups with different symbols (†, ‡), *p* < 0.0001. HPF = high‐power field. Symbols (*, †, ‡) indicate significance for each other (at 0.05 level). All statistical analyses were performed by one‐way anova, followed by Bonferroni multiple comparison post hoc tests (*n* = 6 for each group). H_2_O_2_, hydrogen peroxide; ADMSCs, adipose‐derived mesenchymal stem cells; PrPc^OE^‐ADMSCs, overexpression of cellular prion protein in ADMSCs; LPS, lipopolysaccharide

Under the oxidative stress, that is treated by H_2_O_2_, the ADMSCs migratory ability was significantly lower in G2 than in G1 and G3 and significantly lower in G3 than in G1 (Figure 2A–G). Additionally, under the inflammatory stimulation, that is treated by LPS, the ADMSCs migratory ability also exhibited an identical pattern of oxidative stress among groups G4 to G6 (Figure 2H–N). These findings implied that PrPc gene overexpression in ADMSCs offered much more excellent benefits than the ADMSC‐only counterpart in maintaining the migratory cell ability against oxidative stress or inflammatory stimulation.

### 3.3 Impact of PrPc^OE^‐ADMSCs on suppressing the inflammatory expression of macrophage cell line and the circulatory level of proinflammatory cytokines

**FIGURE 3:**
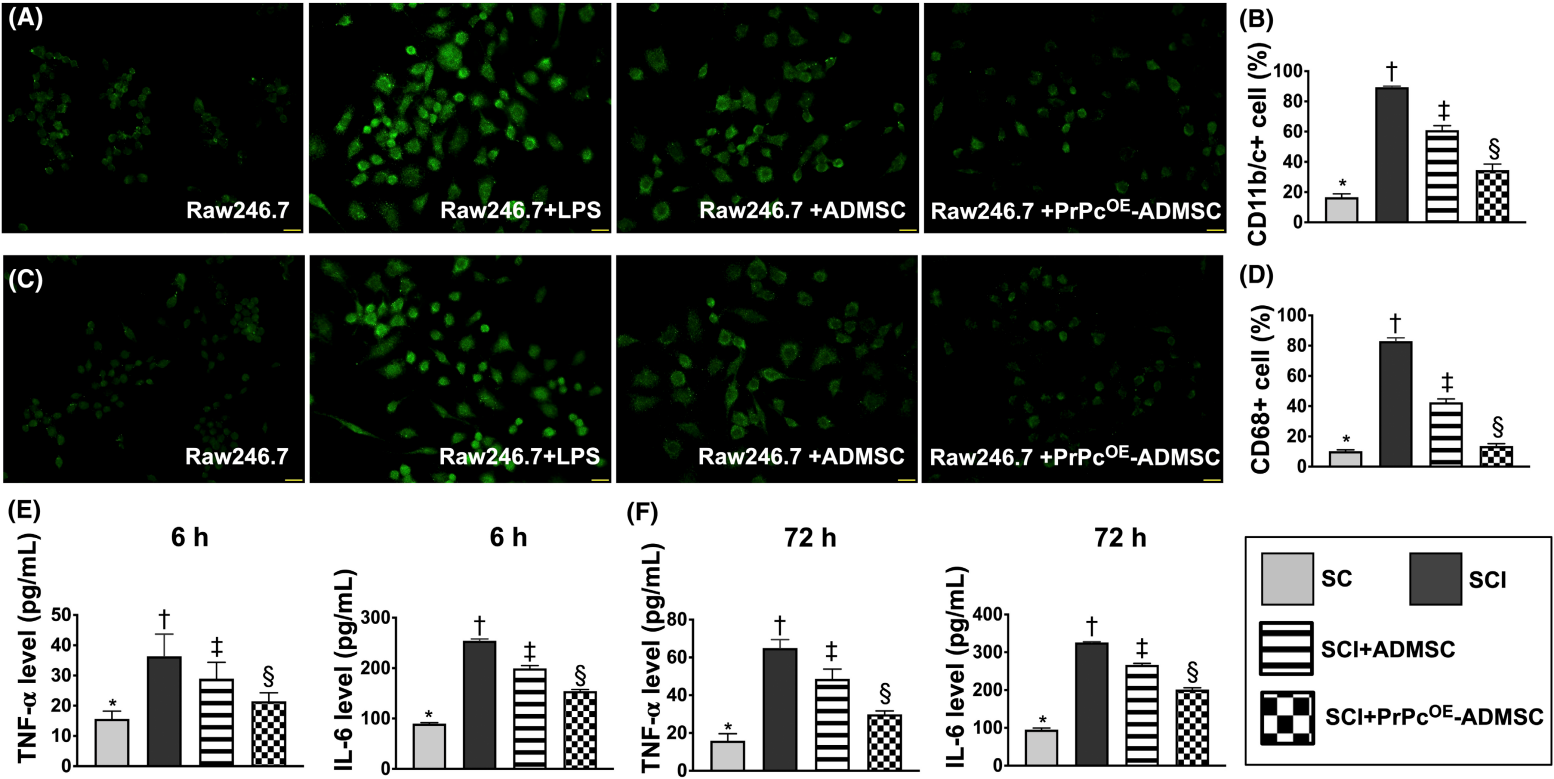
Impact of PrPc^OE^‐ADMSCs on suppressing the inflammatory expression of macrophage cell line and the circulatory levels of inflammatory cytokines. (A) Illustrating the immunofluorescent (IF) microscopic finding (400x) for identification of CD11b positively stained surface marker in Raw 264.7 cells (i.e. a macrophage cell line) (green colour). (B) Analytical result of the number of CD11b + cells. (C) Illustrating the IF microscopic finding (400x) for identification of CD68 positively stained surface marker in Raw 264.7 cells (green colour). (D) Analytical result of the number of CD168+ cells. Scale bars in the right lower corner represent 20 μm. (E) Circulating levels of tumour necrosis factor (TNF)‐α and interleukin (IL)‐6 at 6 h after SCI procedure. (F) Circulating level of TNF‐α and IL‐6 at 72 h after SCI procedure. * vs. other groups with different symbols (†, ‡, §), *p* < 0.0001. *n* = 4 for each group. All statistical analyses were performed by one‐way anova, followed by Bonferroni multiple comparison post hoc test. Symbols (*, †, ‡, §) indicate significance for each other (at 0.05 level). ADMSCs, adipose‐derived mesenchymal stem cells; PrPc^OE^‐ADMSCs, overexpression of cellular prion protein in ADMSCs; SC, sham‐operated control; SCI, spinal cord injury

In the in vitro study, we utilized the Raw 264.7 cells (i.e. a macrophage cell line) to assess the therapeutic impact of PrPc^OE^‐ADMSCs on suppressing the cellular level of inflammatory expression and the cells, therefore, were categorized into R1 (Raw 264.7), R2 (Raw 264.7 + LPS/50 ng/ml for 6 h treatment), R3 (Raw 264.7 + LPS + ADMSCs), and R4 (Raw 264.7 + LPS + PrPc^OE^‐ADMSC). The IF microscopic finding demonstrated that the surface marker expressions of CD11b and CD68 in Raw 264.7 cells, two indicators of inflammation, were highest in R2, lowest in R1 and significantly higher in R3 than R4 (Figure 3A–D).

Next, we collected the circulatory blood sample from post SCI rats and utilized the ELISA to determine the circulating levels of TNF‐α and IL‐6. The results showed that these two parameters were highest in group 2 (SCI), lowest in group 1 (SC), and significantly higher in group 3 (SCI + ADMSCs) than in group 4 (SCI + PrPc^OE^‐ADMSCs) at time points of 6 h and 72 h after SCI induction in rodent (Figure 3E,F). Our in vitro and in vivo studies indicated that PrPc^OE^‐ADMSCs were better than merely ADMSCs alone at attenuating the inflammatory reaction.

### 3.4 The time courses of neurological function after acute SCI procedure

**FIGURE 4:**
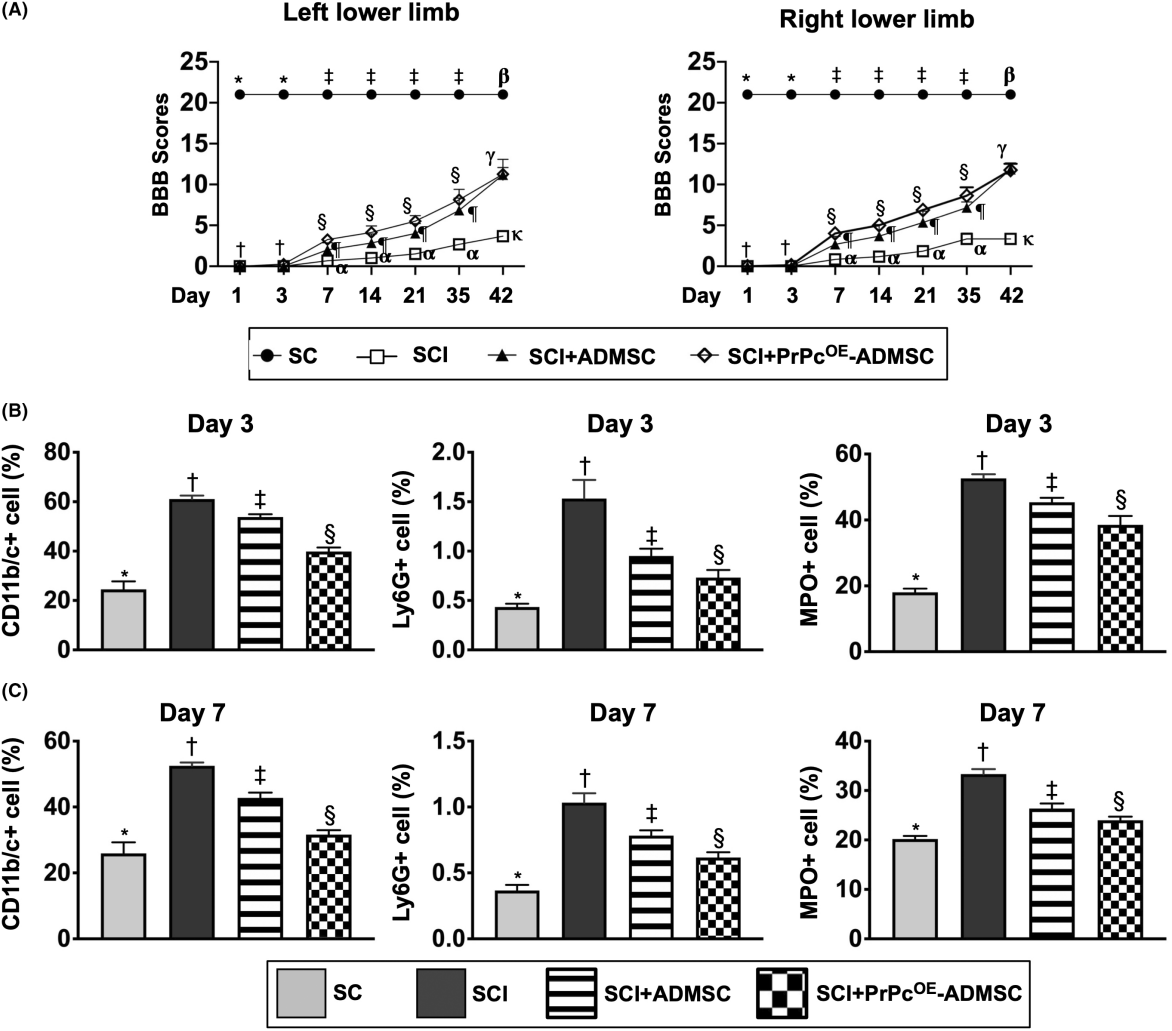
Time courses of BBB scores (i.e. an indicator of neurological function) and circulatory inflammatory cells after acute SCI procedure. (A) By Days 1 and 3: * vs. †, *p* < 0.0001; symbol † indicated the *p* value >0.5 among the groups of SC, SCI only, SCI + ADMSCs and SCI + PrPc‐ADMSCs at these time points with respect to both right and left lower limbs. By Days 7, 14, 21 and 35, ‡ vs. other groups with different symbols (§, ¶, α), *p* < 0.0001 at these time points with respect to both right and left lower limbs. By day 42, ß vs. other groups with different symbols (γ, κ), *p* < 0.0001 at these time points with respect to both right and left lower limbs; γ indicated the *p* value >0.5 between the groups of SCI + ADMSCs and SCI + PrPc‐ADMSCs at this time point (i.e. by Day 42). All statistical analyses were performed by one‐way anova, followed by Bonferroni multiple comparison post hoc test (*n* = 8 for each group). Symbols (‡, §, ¶, α) or (ß, γ, κ) indicate significance for each other (at 0.05 level). (B‐C) Flow cytometric analysis of circulatory inflammatory cells (CD11b/c+, Ly6G+, myeloperoxidase (MPO)+) by Days 3 and 7 after acute SCI procedure. * vs. other groups with different symbols (†, ‡, §), *p* < 0.0001. All statistical analyses were performed by one‐way anova, followed by Bonferroni multiple comparison post hoc test (*n* = 6 for each group). Symbols (*, †, ‡, §) indicate significance for each other (at 0.05 level). ADMSCs, adipose‐derived mesenchymal stem cells; PrPc^OE^‐ADMSCs, overexpression of cellular prion protein in ADMSCs; BBB score, Basso, Beattie, Bresnahan score; SC, sham‐operated control; SCI, spinal cord injury

The BBB functional scale for assessing the locomotor capacity of rats was performed for each rat on Days 1, 3, 7, 14, 21, 35 and 42 after acute SCI induction (Figure 4A). By Days 1 and 3, the BBB score of the left or right lower limb was significantly higher in group 1 than in groups 2, 3 and 4, but it did not differ among groups 2–4 at these time intervals. Additionally, by days 7–42, this parameter remained persistently higher in group 1 than that of groups 2–4 with respect to either left or right lower limb. However, as compared with group 2, this parameter of left or right lower limb was significantly higher in groups 3 and 4 and significantly higher in group 4 than in group 3 at the time points of Days 7–35, whereas this parameter did not differ between groups 3 and 4 by the time point of day 42 (i.e., the end of the study period). These findings implicated the attainment of a steady‐state of neurological functional improvement after Days 7–42 following SCI procedure between groups 3 and 4 animals as compared with group 2 animals. Additionally, by the time point of Day 42, the ADMSCs were non‐inferior to PrPc^OE^‐ADMSCs for improving the BBB score, suggesting that it could be attributed to the intrinsic capacity of recovery in neurological function in rodent. Taken together, both ADMSCs and PrPc^OE^‐ADMSCs significantly improved neurological functions compared to SCI group 2.

### 3.5 Flow cytometric analysis of circulatory inflammatory cells by Days 3 and 7 after acute SCI procedure

To elucidate the circulatory levels of inflammatory cells at the acute phase of SCI, the flow cytometric analysis was utilized in the present study. Consistent with in vitro study, the circulatory levels of CD11b/c+, Ly6G+ and MPO+ cells, three indicators of inflammation, were lowest in group 1, highest in group 2 and significantly higher in group 3 than in group 4 at the time points of Days 3 and 7 after acute SCI procedure (Figure 4B,C), suggesting that PrPc^OE^‐ADMSCs might have better ability than ADMSCs on suppressing the acute inflammatory reaction.

### 3.6 Role of NLRP3 inflammasome signalling in the acute phase (i.e. by Day 3) of SCI

**FIGURE 5:**
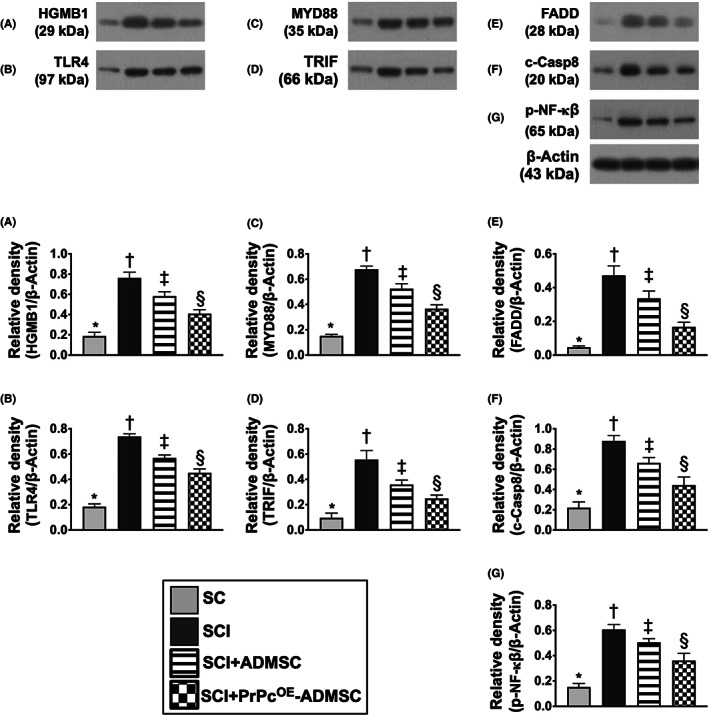
Role of upstream NLRP3 inflammasome signalling in the acute phase (i.e. by Day 3) of SCI. (A) Protein expression of High mobility group box 1 (HMGB1). (B) Protein expression of Toll‐Like Receptor 4 (TLR4). (C) Protein expression of myeloid differentiation primary response 88 (MyD88). (D) Protein expression of TIR‐domain‐containing adapter‐inducing interferon‐β (TRIF). (E) Protein expression of FAS‐associated death domain (FADD). (F) Protein expression of cleaved caspase8 (c‐Casp8). (G) Protein expression of phosphorylated (p)‐nuclear factor kappa B (p‐NF‐κB), * vs. other groups with different symbols (†, ‡, §), *p* < 0.0001. All statistical analyses were performed by one‐way anova, followed by Bonferroni multiple comparison post hoc test (*n* = 6 for each group). Symbols (*, †, ‡, §) indicate significance for each other (at 0.05 level). ADMSCs, adipose‐derived mesenchymal stem cells; PrPc^OE^‐ADMSCs, overexpression of cellular prion protein in ADMSCs; SC, sham‐operated control; SCI, spinal cord injury

**FIGURE 6:**
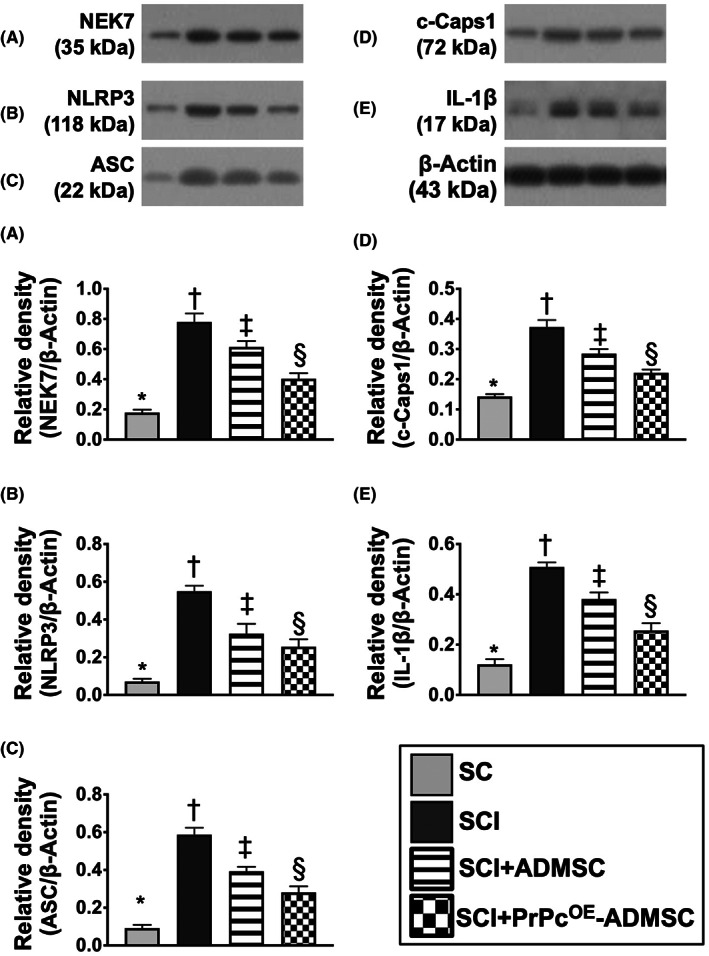
Role of downstream NLRP3 inflammasome signalling in acute phase (i.e. by Day 3) of SCI. (A) Protein expression of NIMA related kinase 7 (NEK7). (B) Protein expression of NLR family pyrin domain containing 3 (NLRP3). (C) Protein expression of apoptosis‐associated speck‐like protein containing a caspase recruitment domain (ASC). (D) Protein expression of cleaved caspase 1 (c‐Csp1). (E) Protein expression of interleukin (IL)‐1ß, * vs. other groups with different symbols (†, ‡, §), *p* < 0.0001. All statistical analyses were performed by one‐way anova, followed by Bonferroni multiple comparison post hoc test (*n* = 6 for each group). Symbols (*, †, ‡, §) indicate significance for each other (at 0.05 level). ADMSCs, adipose‐derived mesenchymal stem cells; PrPc^OE^‐ADMSCs, overexpression of cellular prion protein in ADMSCs; SC, sham‐operated control; SCI, spinal cord injury

**FIGURE 7:**
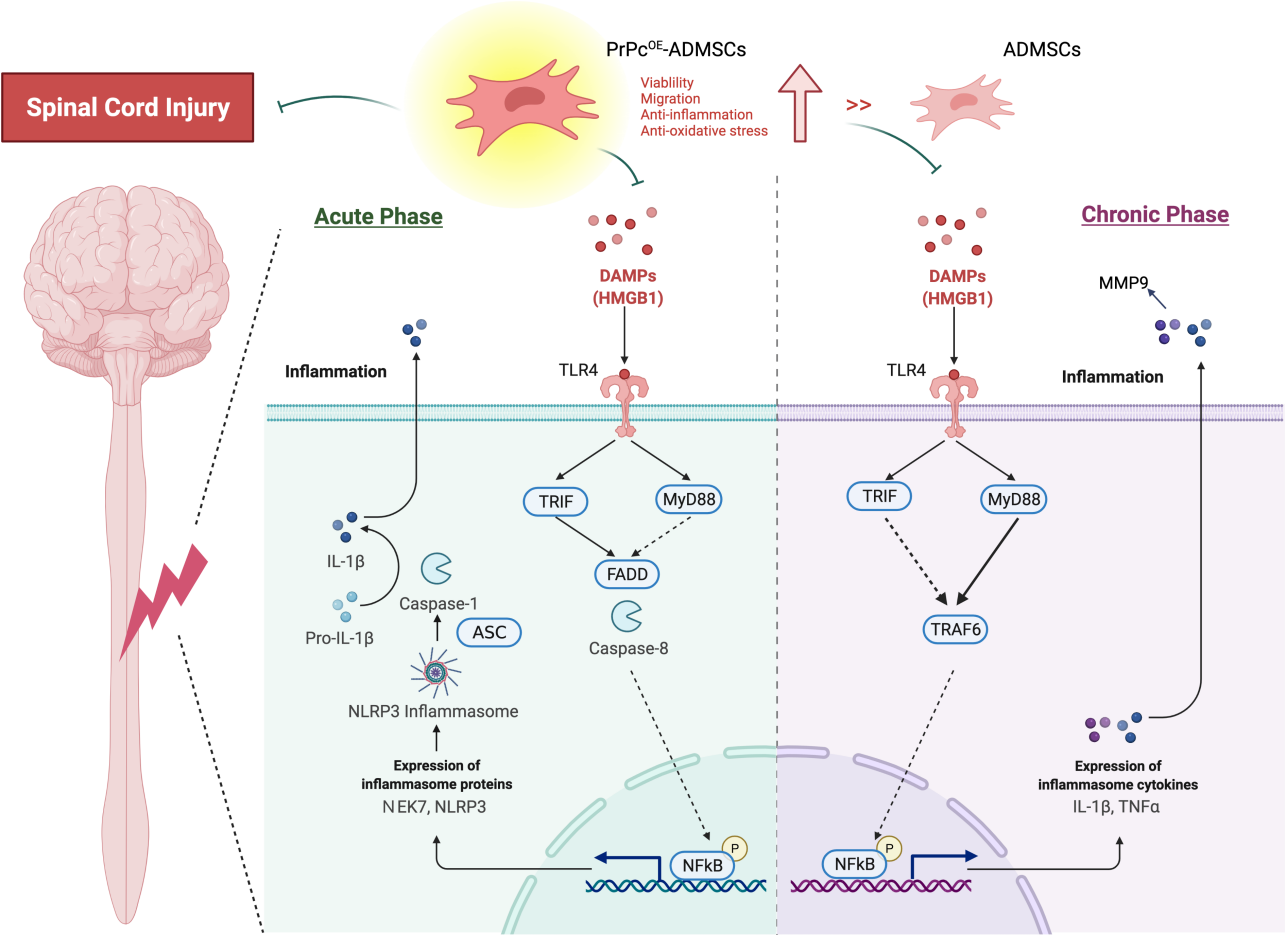
Schematically illustrated the proposed mechanism of NLRP3 inflammasome signalling participated in the acute and chronic phases of SCI. ADMSCs, adipose‐derived mesenchymal stem cells; PrPc^OE^‐ADMSCs, overexpression of cellular prion protein in ADMSCs; SCI, spinal cord injury

To delve into the essential role of NLRP3 inflammasome signalling on neurological dysfunction in the setting of SCI, six animals in each group were euthanized and the Western blot analysis was utilized by Day 3 after the SCI procedure. The result demonstrated that the protein expressions of HGB1, TLR4, MyD88, TRIF, FADD, cleaved caspase8 and p‐NF‐κB (Figure 5), seven biomarkers of upstream inflammasome signalling, and the protein expressions of NEK7, NLRP3, ASC, cleaved caspase1 and IL‐1ß (Figure 6), five indicators of downstream inflammasome signalling, were lowest in group 1, highest in group 2 and significantly higher in group 3 than in group 4. Additionally, the protein expression of PrPc was highest in group 1, lowest in group 2 and significantly higher in group 4 than in group 3. These findings suggested that intrinsic PrPc would be suppressed in the spinal cord in the acute phase of SCI. The PrPc^OE^‐ADMSCs were better than ADMSCs only for inhibiting the activation of NLRP3 inflammasome signalling. Based on our findings, we schematically proposed the underlying mechanism of the PrPc^OE^‐ADMSCs might be superior to ADMSCs therapy in attenuating the expression of NLRP3 inflammasome signalling in the acute phase of SCI in rodents (Figure 7).

### 3.7 Role of DAMP‐signalling inflammatory pathway in chronic phase (i.e. by Day 42) of SCI

To deeply uncover the crucial role of the DAMP signalling pathway on neurological damage in the setting of SCI, six animals in each group were euthanized and the Western blot analysis was utilized again by Day 42 after the SCI procedure. The result showed that the protein expressions of HGMB1, TLR4, MyD88, TRIF, TRAF6 and p‐NF‐κB, six indicators of upstream DAMP‐inflammatory signalling, and the protein expressions of TNF‐α, MMP‐9 and IL‐1ß, three indicators of downstream inflammatory DAMP signalling, were highest in group 2, lowest in group 1 and significantly higher in group 3 than in group 4 (Figure S1). Additionally, the protein expression of PrPc was highest in group 1, lowest in group 2 and significantly higher in group 4 than in group 3. These finding implicated that the PrPc^OE^‐ADMSCs was better than ADMSCs merely for ameliorating the activation of DAMP‐inflammatory signalling. Based on these findings, we schematically proposed the underlying mechanism of the PrPc^OE^‐ADMSCs might be superior to ADMSCs therapy in attenuating the expression of the DAMP‐inflammatory signalling pathway in the chronic phase of SCI in rodent (Figure 7).

### 3.8 Gene and protein expressions of PrP^C^ in ADMSC and the expression of MSCs surface markers

The relative gene expression and protein expression of PrP^C^ in PrPc^OE^‐ADMSCs was significantly increased than in ADMSCs, suggesting that PrP^C^ overexpression in ADMSCs was successful conducted (Figure S2A,B). Additionally, the flow cytometric analysis showed that the ADMSCs surface markers, including CD29, CD73 and CD99 were highly populated by day‐14 cell culture (Figure S2C–E).

## 4 DISCUSSION

This study which investigated the therapeutic impact of PrPc^OE^‐ADMSCs on protecting the integrity of the spinal cord against acute SCI in rodents yielded several striking implications. First, the present study results observed that the NLRP3 inflammasome and DAMP‐inflammatory signalling pathways might play important roles in the damage of the spinal cord in the setting of SCI. Second, PrPc^OE^‐ADMSCs were better than ADMSCs merely in early improving the neurological function in rats after acute SCI. Third, as compared with ADMSCs, the PrPc^OE^‐ADMSCs owned a greater capacity to inhibit the inflammatory reaction and generation of oxidative stress, resulting in protecting the spinal cord against acute SCI.

Interestingly, our previous studies have shown that ADMSCs therapy has a strong capacity of downregulating the inflammation and oxidative stress in many different disease entities.^22^, ^23^, ^24^, ^25^, ^26^ The previous study indicated MSC therapy facilitated the secretion of the soluble antioxidant to reduce oxidative insults in SCI.^27^ Additionally, our recent study has further demonstrated that PrPc^OE^‐ADMSCs have a remarkable ability to attenuate inflammatory activity and oxidative stress.^35^ An essential finding in the in vitro studies of the present study was that as compared with those of ASMSCs the PrPc^OE^‐ADMSCs offer more greater capacity for ameliorating H_2_O_2_‐induced oxidative stress and LPS‐induced inflammatory reaction. In this way, our findings strengthened the findings of our previous studies.^22^, ^23^, ^24^, ^25^, ^26^

Originally, PrPc was identified as a glycosylphosphatidylinositol‐anchored glycoprotein that was enriched in the brain and nerve cells and also displayed in other tissues.^38^, ^39^ Subsequently, the PrPc, acting as a neuroprotective or survival protein for cell proliferation and growth, has been discovered because this cellular protein prevents Bcl‐2‐associated protein X (Bax)‐mediated cell apoptosis and death.^40^ To further prove these doctrines,^38^, ^39^, ^40^ we performed another in vitro study. Our result demonstrated that as compared with ADMSCs only, the cell viability/proliferation rate and migratory ability were much better in PrPc^OE^ in ADMSCs. Our above‐mentioned in vitro studies and the findings of previous studies^38^, ^39^, ^40^ encouraged us to perform an acute SCI animal model by using the PrPc^OE^ in ADMSCs therapy.

The most important finding of the present study was that as compared with the SCI animals, the BBB score (i.e. an index of recovery of neurological function) was substantially preserved in that of SCI animals after receiving ADMSCs treatment. Interestingly, our previous studies have clearly identified that ADMSCs therapy significantly reduced brain infarct size and preserved neurological function in rodents after acute ischemic stroke.^41^, ^42^ In this way, our finding was comparable with that of our previous studies.^41^, ^42^ Of distinctive finding was that PrPc^OE^‐ADMSCs therapy was found to be better than ADMSCs therapy for the improvement of BBB score at the early and convalescent stages of SCI. However, this advantage was lost at the chronic phase of SCI, suggesting that it could be attributed to the admirably inherent capacity of rodents for self‐regeneration. The other cause may also be because one dosage of PrPc^OE^‐ADMSCs therapy is not enough when further improvement of neurological function is considered in the chronic phase of SCI.

In response to the distinctive outcome of neurological functional recovery was identified, the next step had to perform the bench works to delineate the possible underlying mechanism of these promising treatments. Intriguingly, when carefully reviewing the previous studies, we could find that acute tissue/organ injury always elicits vigorous inflammatory reaction.^31^, ^32^, ^33^, ^35^, ^41^, ^42^ By acute phase after acute SCI procedure, we found the circulatory levels of inflammatory cells (i.e. by flow cytometry) and proinflammatory cytokines (i.e. by ELISA) were markedly increased in animals with SCI injury, suggesting that systemically inflammatory reaction was elicited in the setting of SCI. Additionally, when looking at the molecular level (i.e. Western blot analysis), we found the NLRP3 inflammasome was substantially upregulated in SCI animals. In addition to corroborating the findings of previous studies,^31^, ^32^, ^33^, ^35^, ^41^, ^42^ our findings indicated that this acute inflammatory signalling pathway might play a crucial role in the damage of the spinal cord after SCI induction. An important finding was that PrPc^OE^‐ADMSCs were superior to ADMSCs only in suppressing upstream and downstream inflammatory signalling.

Further, we were curious about the inflammatory reaction and what the crucial inflammatory signalling in the chronic phase (i.e., by day 42 after SCI induction) of SCI was, the Western blot analysis was again utilized. As we expected, not only one inflammatory signalling (i.e. NLRP3 inflammasome) but also the DAMP inflammatory signalling biomarkers were found remaining to be notably increased, suggesting that the chronic inflammatory reaction could be persistently present after SCI for the destruction of the neurons and tissues, resulting in impeding the neurological functional recovery in a distant future. Of promising findings was that these two inflammatory signalling were markedly suppressed by ADMSCs and further markedly suppressed by PrPc^OE^‐ADMSCs, highlighting that this strategic management, particularly in the utilization of PrPc, which is the critical protein for neuron growth and proliferation, could be considered for the SCI patients, especially when they are refractory to conventional treatment.

### 4.1 Study limitation

This study has limitations. First, although the study period was 42 days, we remain concerned about whether such a time interval was long enough to define a true ‘chronic phase’ of SCI. Second, we did not test whether two doses of cell therapy would be better than a single dose for improving the outcome in the setting of SCI. Third, we remain uncertain whether two doses of PrPc^OE^ in ADMSCs would be superior to two doses of ADMSCs for augmenting the recovery of neurological function. Finally, we did not utilize agonists or antagonists (i.e. such as drugs, specific inhibitors, or gene manipulations) to prove or disprove the proposed signalling pathways of NLRP3 inflammasome and DAMP‐inflammation involved in SCI. Thus, we cannot rule out there may also have other signalling pathways to participate in the damage of the injured spinal cord.

## 5 CONCLUSION

Overall, inflammatory signalling might play a fundamental role in spinal cord damage and retardation of neurological functional recovery. PrPc^OE^‐ADMSCs might be superior to AMDSCs in improving the outcomes after acute SCI.

## AUTHOR CONTRIBUTIONS

**Tsung‐Cheng Yin:** Funding acquisition (lead); investigation (equal); validation (equal); visualization (equal). **Yi‐Chen Li:** Formal analysis (equal); visualization (equal); writing – review and editing (equal). **Pei‐Hsun Sung:** Formal analysis (equal); methodology (equal). **John Y. Chiang:** Formal analysis (equal); writing – review and editing (equal). **Pei‐Lin Shao:** Methodology (equal); validation (equal). **Hon‐Kan Yip:** Conceptualization (lead); supervision (equal); writing – original draft (lead); writing – review and editing (equal). **Mel Lee:** Conceptualization (equal); funding acquisition (equal); investigation (lead); supervision (equal); writing – review and editing (equal).

## CONFLICT OF INTEREST

The authors have no conflict of interest.

## Supporting information


Appendix S1

## Data Availability

Data available on request from the authors.
