# Supplementary material for: Adipose‐derived mesenchymal stem cells overexpressing prion improve outcomes via the NLRP3 inflammasome/DAMP signalling after spinal cord injury in rat
Source: J Cell Mol Med. 2023 Jan 20;27(4):482–95. doi: 10.1111/jcmm.17620 (PMC9930430; doi:10.1111/jcmm.17620)
Supplement: Supplementary file 1 — Appendix S1 [file JCMM-27-482-s001.docx]

**SUPPLEMENTARY INFORMATION**

| **Target Protein** | **Antibody** |
| --- | --- |
| Tumor necrosis factor (TNF)-α | 1:1000, Cell Signaling |
| Interleukin (IL)-1ß | 1:1000, Cell Signaling |
| Matrix metalloproteinase (MMP)-9 | 1: 1000, Millipore |
| High mobility group box 1 (HGB1) | 1:1000, Cell Signaling |
| Toll-like receptor 4 (TLR4) | 1:1000, Novus |
| Myeloid differentiation primary response 88 (MyD88) | 1:1000, Abcam |
| TIR-domain-containing adapter-inducing interferon-β (TRIF) | 1:1000, Abcam |
| FAS-associated death domain (FADD) | 1:1000, Abcam |
| cleaved caspase 8 | 1:1000, Cell Signaling |
| phosphorylated (p)-nuclear factor kappa B (p-NF-κB) | 1:1000, Abcam |
| NIMA related kinase 7 (NEK7) | 1:1000, Abcam |
| NLR family pyrin domain containing 3 (NLRP3) | 1:1000, Abcam |
| Apoptosis-associated speck-like protein containing a caspase recruitment domain (ASC) | 1:1000, Abcam |
| cleaved caspase 1 | 1:1000, Novus |
| TNF receptor associated factor 6 (TRAF6) | 1:1000, Abcam |

**Supplementary Table 1. Antibodies list for Western blotting.**

**
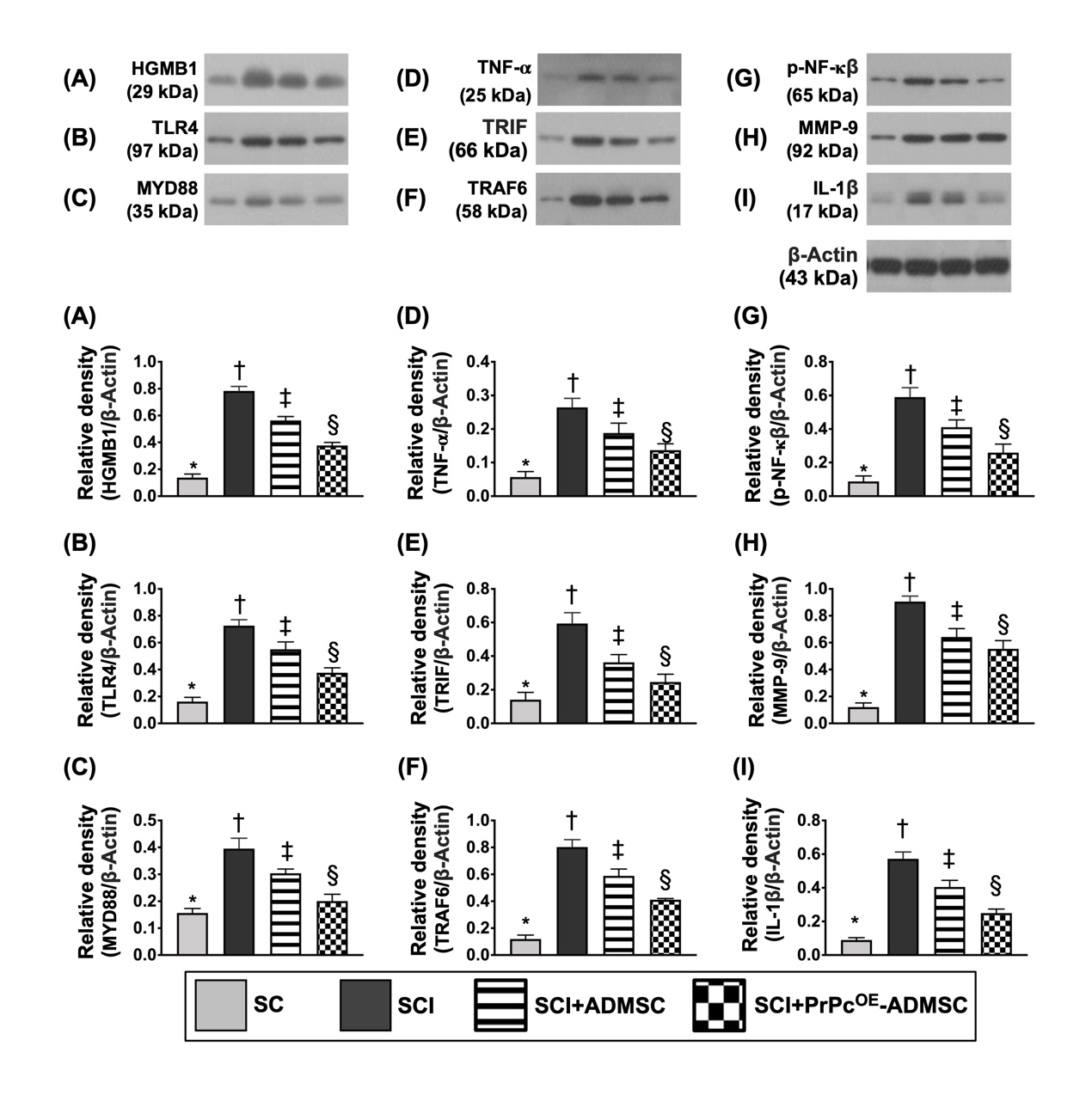
**

**Supplementary Figure 1.** **Role of DAMP-inflammatory signaling in chronic phase (i.e., by day 42) of SCI.** (A) Protein expression of High mobility group box 1 (HMGB1). (B) Protein expression of Toll-Like Receptor 4 (TLR4). (C) Protein expression of myeloid differentiation primary response 88 (MyD88). (D) Protein expressions of tumor necrosis factor alpha (TNF-α). (E) Protein expression of TIR-domain-containing adapter-inducing interferon-β (TRIF). (F) Protein expression of TNF receptor associated factor 6 (TRAF6). (G) Protein expression of phosphorylated nuclear factor kappa B (p-NF-κB). (H) Protein expression of matrix metalloproteinase (MMP)-9. (I) Protein expression of interleukin (IL)-1ß, * vs. other groups with different symbols (†, ‡, §), p<0.0001. All statistical analyses were performed by one-way ANOVA, followed by Bonferroni multiple comparison post hoc test (n = 6 for each group). Symbols (*, †, ‡, §) indicate significance (at 0.05 level). SC = sham-operated control; SCI =spinal cord injury; ADMSCs = adipose-derived mesenchymal stem cells; PrPc^OE^-ADMSCs = overexpression of cellular prion protein in ADMSCs.


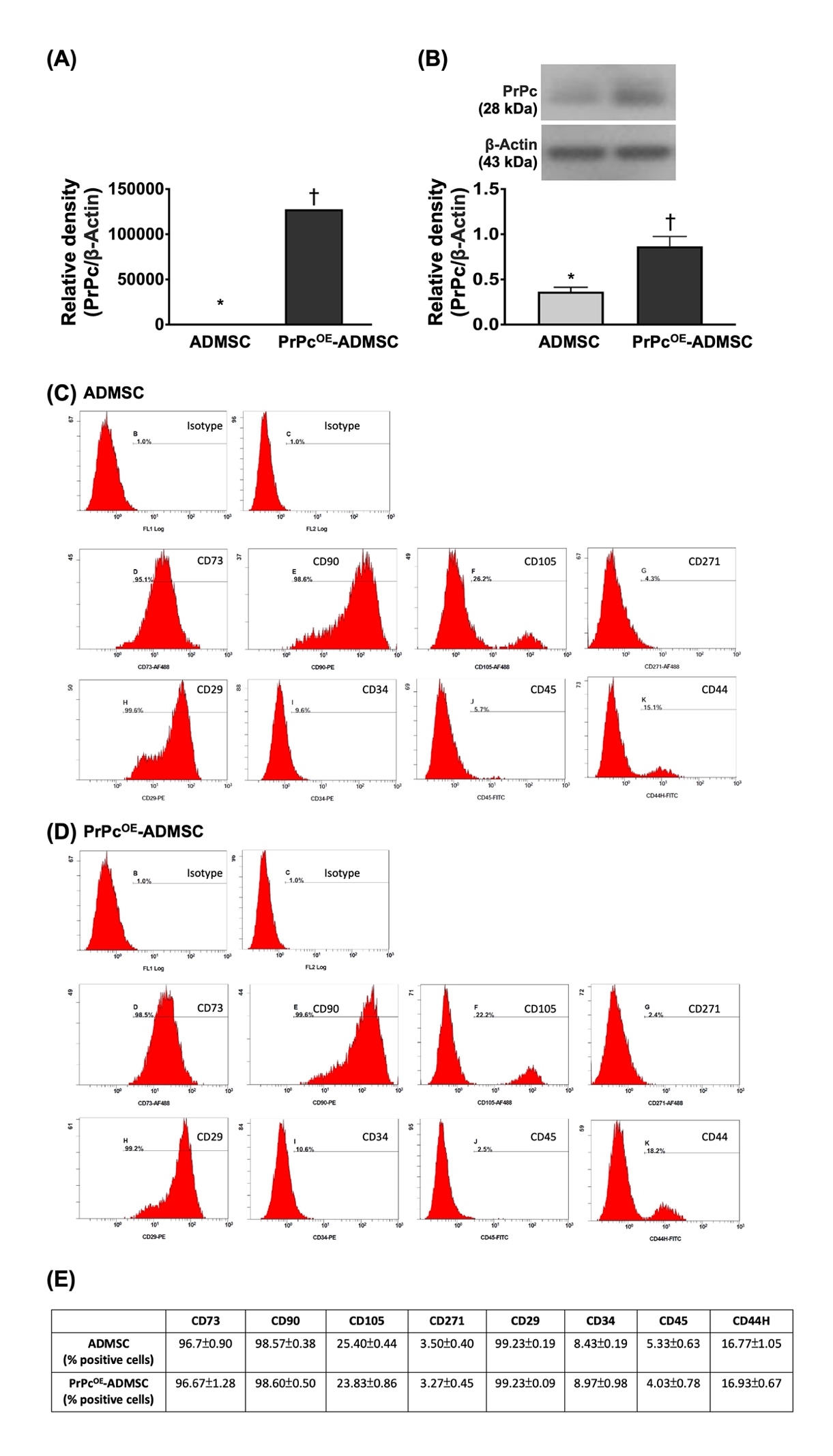


**Supplementary Figure 2.** **Gene and protein expressions of PrP^C^ in ADMSC and the expression of MSCs surface markers after 14-day cell culture**

**A)** qPCR result showed that the relative gene expression of PrP^C^ in PrPc^OE^-ADMSCs was significantly increased than in ADMSCs, * vs. †, p<0.001. **B)** The protein expression of PrP^C^, * vs. †, p<0.001. **C)** Illustrating the flow cytometric analysis for identification of the population of MSCs surface markers at day-14 ADMSC culturing. **D)** Illustrating the flow cytometric analysis for identification of the population of MSCs surface markers by day-14 PrPc^OE^-ADMSCs culturing. **E)** The Table was listed the percentage of different MSCs surface markers in AMDCs group and PrPc^OE^-ADMSCs group after 14-day cell culturing. Note that adipose tissue giving rise to ADMSC in culture had a low population of heterogeneous CD34 and CD45 phenotype, i.e., hematopoietic surface markers. n = 3 per each group.
